# The blanking interval after atrial fibrillation ablation: time for reassessment with pulsed field energy—Authors’ reply

**DOI:** 10.1093/europace/euag165

**Published:** 2026-07-22

**Authors:** Juan F Rodriguez-Riascos, Hema S Vemulapalli, James Y Kim, Luis R Scott, Arturo M Valverde, Mayank Sardana, Abhishek J Deshmukh, Suraj Kapa, Chris McLeod, Komandoor Srivathsan

**Affiliations:** Division of Cardiovascular Diseases, Mayo Clinic, 5777 East Mayo Boulevard, Phoenix, AZ 85054, USA; Division of Cardiovascular Diseases, Mayo Clinic, 5777 East Mayo Boulevard, Phoenix, AZ 85054, USA; Department of Internal Medicine, Harlingen Medical Center, Harlingen, TX, USA; Department of Internal Medicine, Mayo Clinic, Phoenix, AZ, USA; Division of Cardiovascular Diseases, Mayo Clinic, 5777 East Mayo Boulevard, Phoenix, AZ 85054, USA; Division of Cardiovascular Diseases, Mayo Clinic, 5777 East Mayo Boulevard, Phoenix, AZ 85054, USA; Division of Cardiovascular Diseases, Mayo Clinic, 5777 East Mayo Boulevard, Phoenix, AZ 85054, USA; Division of Cardiovascular Diseases, Mayo Clinic, Rochester, MN, USA; Division of Cardiovascular Diseases, Mayo Clinic, Rochester, MN, USA; Division of Cardiovascular Diseases, Mayo Clinic, Jacksonville, FL, USA; Division of Cardiovascular Diseases, Mayo Clinic, 5777 East Mayo Boulevard, Phoenix, AZ 85054, USA

We thank the authors for their thoughtful comments on our work. We agree that the clinical implications of early post-ablation arrhythmias must be interpreted with caution as pulsed field ablation (PFA) continues to evolve across diverse patient populations, procedural approaches, and management strategies. However, we wish to clarify a central point: our study does not advocate abandoning the blanking period, nor does it suggest that every early recurrence warrants prompt re-ablation.^1^ Rather, our findings indicate that the traditional interpretation of the blanking period—largely derived from experience with thermal ablation—may require reassessment in the PFA era.^2^

This need for reassessment stems from the distinct mechanism of PFA. Compared with thermal ablation, the inflammatory and healing response following electroporation appears different. Prior histopathological data suggest that inflammation begins to resolve early and is progressively replaced by fibrosis within the initial post-ablation period.^3,4^ Accordingly, arrhythmias occurring beyond the very early phase—particularly after 30 days—may be less likely to reflect transient inflammation and more likely to carry prognostic significance. Our study was designed to evaluate this hypothesis empirically by examining 30-, 60-, and 90-day thresholds following PFA.

We fully acknowledge the heterogeneity in rhythm monitoring as a limitation. To address this, we reported outcomes stratified by monitoring strategy and conducted subgroup analyses in patients with implanted or external rhythm monitoring. The association between early and late recurrence remained significant in this subgroup, supporting the prognostic relevance of early events even under more intensive surveillance.

Similarly, lesion heterogeneity was explicitly acknowledged. Our study should not be interpreted as demonstrating a uniform PFA-specific healing mechanism across all patients. Our objective was primarily prognostic rather than mechanistic. Notably, the association between early and late recurrence remained consistent across clinically relevant subgroups, including patients with paroxysmal and non-paroxysmal atrial fibrillation, as well as those undergoing first-time and redo ablation. This consistency supports the broader clinical relevance of early recurrence as a marker of subsequent arrhythmia risk.

We agree that early recurrence does not equate to failed lesion durability, nor does it establish that earlier intervention improves outcomes. Our study was not designed to evaluate whether early cardioversion, antiarrhythmic drug escalation, or repeat ablation improves long-term outcomes following early recurrence. We emphasized the importance of shared decision-making, with redo ablation reserved for patients in whom symptomatic burden, thromboembolic risk, or atrial remodelling outweighs procedural risks. Nevertheless, identifying early recurrence as a high-risk signal may justify closer follow-up, more deliberate rhythm surveillance, and more realistic patient counselling.

Finally, we note that recent consensus trends towards shortening the blanking period to approximately 8 weeks are aligned with evolving evidence. Our findings suggest that even earlier events—including those within 30 days—may carry prognostic significance. In the context of PFA, early recurrence should not be dismissed as a benign inflammatory phenomenon but rather recognized as a clinically meaningful marker of long-term arrhythmia risk, warranting closer follow-up and further investigation. In our view, the available evidence supports reassessing the conventional 90-day framework and considering a shorter interval, while future prospective studies might clarify how timing, burden, pattern, monitoring strategy, and clinical context should be integrated into post-ablation management.

## Data Availability

No new data were generated or analysed in support of this response.
